# Phase II trial of palbociclib in patients with metastatic urothelial cancer after failure of first-line chemotherapy

**DOI:** 10.1038/s41416-018-0229-0

**Published:** 2018-10-08

**Authors:** Tracy L. Rose, David D. Chism, Ajjai S. Alva, Allison M. Deal, Susan J. Maygarden, Young E. Whang, Jordan Kardos, Anthony Drier, Ethan Basch, Paul A. Godley, Mary W. Dunn, William Y. Kim, Matthew I. Milowsky

**Affiliations:** 1 Division of Hematology/Oncology, Lineberger Comprehensive Cancer Center, University of North Carolina at Chapel Hill, Chapel Hill, NC USA; 2 Division of Hematology and Oncology, Department of Medicine, Vanderbilt-Ingram Cancer Center, Vanderbilt University Medical Center, Nashville, TN USA; 3 Division of Hematology/Oncology, University of Michigan, Ann Arbor, MI USA; 4 Lineberger Comprehensive Cancer Center, University of North Carolina at Chapel Hill, Chapel Hill, NC USA; 5 Department of Pathology and Laboratory Medicine, University of North Carolina at Chapel Hill, Chapel Hill, NC USA

**Keywords:** Bladder cancer, Tumour biomarkers, Cancer genomics

## Abstract

**Background:**

The majority of urothelial cancers (UC) harbor alterations in retinoblastoma (Rb) pathway genes that can lead to loss of Rb tumour suppressor function. Palbociclib is an oral, selective inhibitor of CDK 4/6 that restores Rb function and promotes cell cycle arrest.

**Methods:**

In this phase II trial, patients with metastatic platinum-refractory UC molecularly selected for p16 loss and intact Rb by tumour immunohistochemistry received palbociclib 125 mg p.o. daily for 21 days of a 28-day cycle. Primary endpoint was progression-free survival at 4 months (PFS4) using a Simon’s two-stage design. Next-generation sequencing including Rb pathway alterations was conducted.

**Results:**

Twelve patients were enrolled and two patients (17%) achieved PFS4 with insufficient activity to advance to stage 2. No responses were seen. Median PFS was 1.9 months (95% CI 1.8–3.7 months) and median overall survival was 6.3 months (95% CI 2.2–12.6 months). Fifty-eight percent of patients had grade ≥3 hematologic toxicity. There were no *CDKN2A* alterations found and no correlation of Rb pathway alterations with clinical outcome.

**Conclusions:**

Palbociclib did not demonstrate meaningful activity in selected patients with platinum-refractory metastatic UC. Further development of palbociclib should only be considered with improved integral biomarker selection or in rational combination with other therapies.

## Background

Urothelial cancer (UC) is a common malignancy with limited treatment options and poor outcomes in patients with metastatic disease. For decades, platinum-based combination chemotherapy was the only effective treatment option for metastatic UC. More recently, with the FDA approval of several immune checkpoint inhibitors, the landscape of treatment options has broadened significantly. However, immune checkpoint inhibition remains effective in only a subset of patients with metastatic UC, and tumour progression remains inevitable in most patients. Additional effective agents are needed for the treatment of this deadly disease and molecularly targeted therapies hold promise for patients with disease progression after platinum-based chemotherapy and/or immunotherapy. For example, the irreversible ErbB family receptor blocker afatinib had significant activity in patients with platinum-refractory UC with *HER2* or *ERBB3* amplifications.^1^ Additionally, the VEGF2-R antagonist ramucirumab in combination with docetaxel improved progression-free survival (PFS) compared with docetaxel alone in unselected patients with platinum-refractory UC.^2^

The Cancer Genome Atlas (TCGA) has expanded our knowledge of the molecular landscape of urothelial carcinoma and has demonstrated the frequent alteration of retinoblastoma (Rb) pathway genes in UC. Cyclin-dependent kinase inhibitor 2A (*CDKN2A*) alteration is the most common focal deletion in UC and is found in up to 20–30% of tumours.^3,4^ Loss of *CDKN2A* leads to upregulation of cyclin-dependent kinase (CDK) 4 and 6 activity, thus phosphorylating and inactivating the tumour suppressor Rb, leading to cell cycle progression and tumour growth. Other Rb pathway alterations include loss of function mutations in *CDKN1A* (p21) in 9% and amplification of *E2F3* in 12% of tumours. Together, these molecular alterations suggest that therapeutic agents targeted to the Rb pathway may have activity in the treatment of metastatic UC.

Palbociclib is an oral, highly selective inhibitor of CDK 4 and 6. Inhibition of CDK4 and CDK6 acts to restore the tumour suppressor role of Rb and promote cell cycle arrest. Intact Rb is critical to the mechanism of CDK4/6 inhibition in cancer treatment, and CDKN2A loss with intact Rb mechanistically predicts sensitivity to CDK4/6 inhibitors. Preclinical data in bladder cancer cell lines have shown inactivation of RB1 confers resistance and inactivation of *CDKN2A* confers sensitivity to palbociclib.^5^ Several CDK4/6 inhibitors have recently been FDA-approved for metastatic, hormone-receptor-positive breast cancer in combination with hormone therapy with impressive prolongation of PFS observed in patients on these agents.^6,7^ Prior studies in UC have found striking similarities between ER-positive and luminal breast cancers and the luminal subtype of UC, including the discovery of estrogen receptor signaling and enrichment in luminal breast cancer-specific gene signatures and pathways. Given these similarities in gene expression, as well as the overall molecular landscape of UC and preclinical data, CDK4/6 inhibition is a promising treatment strategy for metastatic UC.^8,9^

We hypothesised that palbociclib would demonstrate clinical activity in patients with UC with Rb pathway alterations who had progressed after standard first-line chemotherapy. We therefore conducted this phase II trial of palbociclib in molecularly selected patients with platinum-refractory UC.

## Methods

### Patients

Patients aged ≥18 years with metastatic histologically confirmed UC of the bladder, urethra, ureter, or renal pelvis who had progressed after prior platinum-based chemotherapy in the perioperative or metastatic setting were enrolled. Immunohistochemistry (IHC) was performed on archival tumour tissue and patients were deemed eligible if the tumours were positive for Rb and negative for p16 as determined by the central study genitourinary pathologist (S.J.M.) in a CLIA-certified laboratory at the lead study site (University of North Carolina). Eligible patients had metastatic disease that was not amenable to curative surgery or radiation, including at least one measurable disease site. Perioperative chemotherapy must have been received within 1 year and no more than two prior cytotoxic chemotherapy regimens were allowed. Inclusion criteria included Eastern Cooperative Oncology Group (ECOG) performance status ≤ 2, absolute neutrophil count ≥ 1500/μL, hemoglobin ≥ 8 g/dL, platelets ≥ 75,000/μL, total bilirubin ≤ 1.5 times the institutional upper limit of normal (ULN), AST/ALT ≤ 2.5 times ULN, serum creatinine ≤ 2.5 times ULN, life expectancy > 3 months, and the ability to provide informed consent. Patients were ineligible if they had received a prior CDK4/6 inhibitor, had active brain metastases, were pregnant or breastfeeding, had uncontrolled systemic disease or uncontrolled infection, or were unable to swallow oral medications. All patients provided written informed consent.

### Study design and treatment

This was an open-label, single-arm multicentre (University of North Carolina, University of Michigan, Vanderbilt) phase II trial to evaluate the efficacy of palbociclib in patients with previously treated UC with p16 loss and intact RB by IHC. Patients received palbociclib 125 mg orally once daily with food on a 21 days on/7 days off schedule in 28-day cycles. Cycles were repeated until disease progression, death, or intolerability. All patients were monitored for toxicity by history, physical examination, and complete blood counts and serum chemistry analysis every 2 weeks for the first two cycles, then every 4 weeks. Dosing was held for any grade 3 hematologic toxicity on day 1 of each cycle or any grade 3 nonhematologic toxicity at any time. Dose reductions were allowed for intolerability with initial dose reduction to 100 mg/day and a subsequent dose reduction to 75 mg/day. Palbociclib was permanently discontinued in patients that required <75 mg/day. Dose intensity was calculated as the dose administered during the total time receiving palbociclib divided by the standard dose intensity specified in the protocol.

Radiologic disease evaluation was performed every 8 weeks with assessment of disease response based on RECIST version 1.1. All adverse events from the start of treatment to 28 days after treatment cessation were graded according to the Common Terminology Criteria for Adverse Events (CTCAE), version 4.0.

### Statistical analysis

The primary endpoint was progression-free survival at 4 months (PFS4) defined as time from treatment initiation to disease progression or death due to any cause. A Simon’s two-stage design was planned to allow for early stopping for futility. The null median PFS was assumed to be 2.5 months,^10^ and a 2-month improvement in PFS was felt to be clinically meaningful, translating to PFS4 improvement from historical rate of 33 to 54%, assuming an exponential distribution. The null hypothesis that the true percentage is 33% was tested against a one-sided alternative. In the first stage, 15 patients were planned to be accrued and if there were five or fewer patients with PFS4 the study would be stopped. Otherwise, 21 additional patients were planned to be accrued for a total of 36. This design yields a type I error rate of 0.046 and power of 0.81, when the true percentage of PFS4 is 54%. It was planned to enroll 40 total patients to allow for potential dropouts and nonevaluable patients. Secondary endpoints were overall response rate, median PFS, and median overall survival (OS) estimated by the Kaplan−Meier method. Study and safety analysis included all patients that received at least one dose of palbociclib.

### Biomarker eligibility and analysis

Eligibility criteria included tumour *CDKN2A* loss and intact Rb, assessed via IHC of p16 and RB respectively, at the primary study site. IHC staining for RB using the purified mouse antihuman RB protein (BD Biosciences Clone G3-245) was validated as a sensitive and specific biomarker for molecular alteration in Rb prior to study initiation. For validation, IHC for Rb was performed on 19 unrelated cancer cases and results were confirmed using prior targeted *RB* gene sequencing results. Thresholds for positive and negative staining were assigned based on staining observed in cases with known intact or deleted Rb. IHC for p16 was performed as per institutional protocol on clinical specimens using a Ventana monoclonal antibody for p16, clone E6H4, epitope retrieval 2/10 with commercial positive and negative controls. For RB, positive staining was >5% of tumour cells showing at least moderate nuclear staining or 20% of tumour cells showing at least weak nuclear staining. Staining less than this was considered negative. For p16, positive staining was >70% of cells showing positive, diffuse expression with strong intensity, with both nuclear and cytoplasmic staining. Staining less than this was considered negative.^11,12^ Based on prior sequencing data in UC, it was estimated that 40% of patients would be eligible for the trial, with approximately 100 patients to be screened to accrue the planned 40 patients. Planned exploratory analyses included correlation of the IHC results with high-throughput sequencing results for DNA alterations in the Rb pathway.

Somatic mutation and copy number analysis was performed on available archival formalin-fixed paraffin-embedded tumours for 11 of the 12 enrolled patients. A pathologist assessed each tumour block for percentage of viable tumour using hematoxylin and eosin-stained slides. Targeted exon sequencing was conducted through the UNCSeq pipeline (v8) to analyse nearly 800 genes associated with cancer as previously described.^13^ Samples were sequenced on the illumina platform on NextSeq 500 sequencers using a commercial customised targeted Agilent SureSelect panel (UNCseq v8). The samples were aligned to hg19 with additional viral reads by BWA (0.7.9a), then sorted, indexed, and duplicates removed by biobambam (2.0.33), realigned with ABRA (0.96), and final bams reprocessed by biobambam. QC metrics were generated by Picard 1.92. Germline variants calls were made with ISAAC (2013) and Freebayes (20140826), then filtered to targeted regions with SnpSift (1.3.4). Somatic calls were made by CADABRA (0.96), Strelka (2013), and UNCeqR (0.14), and merged into a VCF. That vcf was annotated with snpEff (3.3), dbSNP (132 via SnpSift), ENSEMBL (via locally developed tool), and ExAC 0.3 (via locally developed tool). Copy number alterations were detected by ADTEx 1.0.4 and postprocessed with locally developed R scripts. Reported analyses are confined to mutations predicted to have a high or moderate impact on protein function through UNCseq. Pathway mutation frequency was calculated based on the number of samples that contained at least one mutation in the gene list associated with that pathway. The cell cycle pathway was represented by the *CCND1*, *CCNE1*, *CDK4*, *CDK6*, *CDKN1A*, *CDKN1B*, *CDKN2A*, *CDKN2B*, *E2F3*, and *RB1*. Statistically significant CNAs (*p* < 0.05) were selected and categorised based on the standard deviation of the gene-associated logR values (amplification ≥ 2, gain ≥ 1 and <2, shallow deletion ≤ −1 and > −2, and deep deletion ≤ −2). Genes and copy number alterations that were previously reported to be significantly mutated in TCGA analysis of UC are reported here, as well as all relevant cell cycle pathway genes.

## Results

### Patient characteristics

**Fig. 1:**
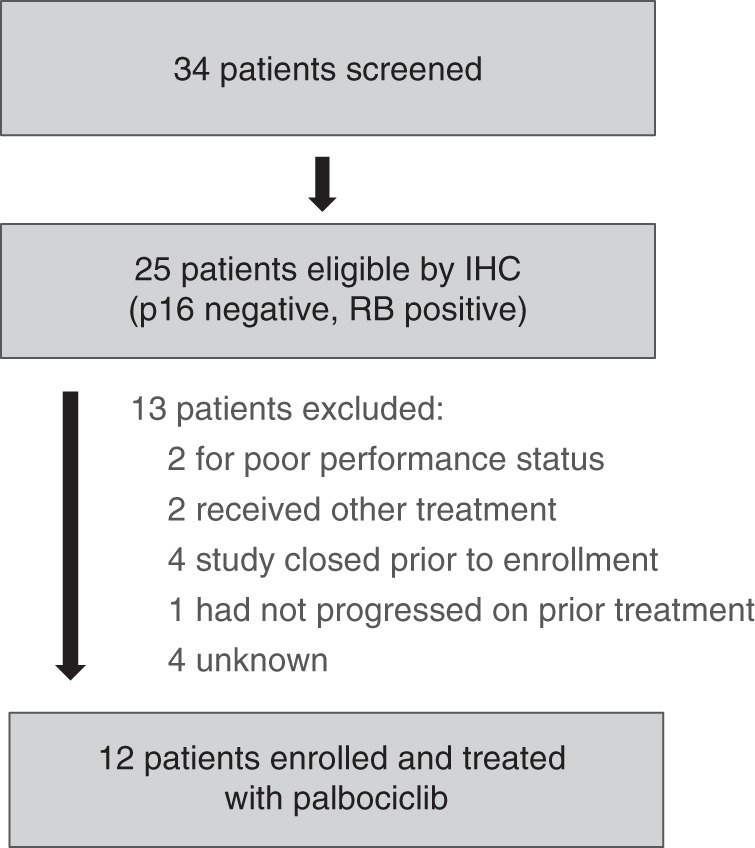
Trial profile

Of 34 patients screened, 25 were eligible based on tumour IHC demonstrating p16 loss and intact Rb (Fig. 1). Of those, 12 patients were enrolled between April 2015 and January 2017. Reasons that eligible patients did not enroll included: decline in performance status (2), received other treatment (2), had not yet progressed on current therapy (1), closure of the study before enrollment could occur (4), and unknown (4).

Baseline patient characteristics are included in Table 1. Most patients were male (67%), age > 65 (67%), and had bladder as the primary site of their UC (58%). Postplatinum prognostic factors included hemoglobin less than 10 g/dL, 17%; liver metastases, 0%; and ECOG performance status more than 0, 58%. All patients received prior platinum-based chemotherapy and ten patients had definitive surgical treatment of their primary tumour (eight had cystectomy, two had nephroureterectomy). Most patients (58%) had only received one prior systemic therapy regimen. Two patients (17%) had received any prior radiation and two patients (17%) had received prior immune checkpoint inhibitor treatment. Three patients (25%) had lymph node-only metastatic disease. Median time from prior chemotherapy to study treatment was 5.0 months.

**Table 1:** Baseline patient characteristics

Characteristic	All, *n* = 12
Age, years	
Median	68
Range	51–86
Sex, *n* (%)	
Female	4 (33.3)
Male	8 (66.7)
Race, *n* (%)	
White	10 (83.3)
Black or African American	2 (16.7)
Ethnicity, *n* (%)	
Hispanic or Latino	1 (8.3)
Non-Hispanic	11 (91.7)
Primary site, *n* (%)	
Bladder	7 (58.3)
Upper tract	4 (33.3)
Urethra	1 (8.3)
Number of prior systemic therapy regimens^a^, *n* (%)	
1	7 (58.3)
2	3 (25.0)
3	2 (16.7)
Setting of prior platinum-based chemotherapy, *n* (%)	
Perioperative only	8 (66.7)
Metastatic only	2 (16.7)
Both	2 (16.7)
ECOG performance status, *n* (%)	
0	5 (41.7)
1	6 (50.0)
2	1 (8.3)
Hemoglobin, *n* (%)	
<10 g/dL	2 (16.7)
≥10 g/dL	10 (83.3)
Liver metastases, *n* (%)	
Yes	0
No	12 (100)
Time from prior therapy, *n* (%)	
<3 months	4 (33.3)
3–6 months	5 (41.7)
6–9 months	1 (8.3)
≥12 months	2 (16.7)

*ECOG PS* Eastern Cooperative Oncology Group performance status

^a^Includes immunotherapy

### Efficacy

Two of the first 12 enrolled patients met the primary endpoint of PFS4 and the best response was stable disease. Since only 2 patients achieved PFS4 in the first 12 patients, it was not possible to meet the criterion for study continuation to stage 2 and thus the study was terminated. The overall response rate was 0% and median PFS was 1.9 months (95% confidence interval 1.8–3.7 months). Median OS was 6.3 months (95% CI 2.2–12.6 months). Eleven of 12 patients have expired; no deaths were felt to be treatment-related. Three patients received subsequent anticancer therapy after protocol treatment.

### Dose intensity and adverse events

The median duration of treatment with palbociclib was 8 weeks (range 2.6–32 weeks). Only four patients were treated beyond cycle 2. The mean dose intensity was 91% and two patients had a dose intensity of <80% (one due to AEs, one due to noncompliance). All patients discontinued the study drug, most commonly due to disease progression (83%). Two patients discontinued the study drug due to adverse events. One patient had a dose reduction due to hematologic toxicity.

Seventy-five percent of study participants experienced grade 3/4 treatment-related AEs and 92% of participants experienced any grade treatment-related AEs (Table 2). Almost all the clinically significant toxicity was hematologic and seven (58%) patients had grade 3 hematologic toxicity (no grade 4). The most common AEs included anemia, leukopenia, lymphopenia, neutropenia, and thrombocytopenia. Serious AEs (any grade) suspected to be related to the study drug did not occur in any patients.

**Table 2:** Overall incidence of AEs and treatment-related AEs

Adverse event	Any grade (*n*, %)	Grade ≥ 3 (*n*, %)
Any AE	12 (100)	11 (92)
Any treatment-related AE	11 (92)	9 (75)
Hematologic AE		
Anemia	9 (75)	3 (25)
Leukopenia	8 (67)	1 (8)
Lymphopenia	8 (67)	2 (17)
Neutropenia	7 (58)	1 (8)
Thrombocytopenia	5 (42)	2 (17)
Lymphocytosis	1 (8)	0
Nonhematologic AE		
Increased creatinine	4 (33)	0
Fatigue	4 (33)	0
Dehydration	2 (17)	1 (8)
Weight loss	2 (17)	0
Alkaline phosphatase increased	2 (17)	0
Anorexia	2 (17)	1 (8)
ALT increased	1 (8)	0
AST increased	1 (8)	0
Constipation	1 (8)	0
Diarrhea	1 (8)	0
Dizziness	1 (8)	0
Dyspnea	1 (8)	0
Hiccups	1 (8)	0
Hyperkalemia	1 (8)	0
Hypocalcemia	1 (8)	0
Hyponatremia	1 (8)	0
Nasal congestion	1 (8)	0
Nausea	1 (8)	0
Sinus tachycardia	1 (8)	0

*AE*adverseevent,*AST*aspartateaminotransferase,*ALT*alanineaminotransferase

### Genomic alterations

**Fig. 2:**
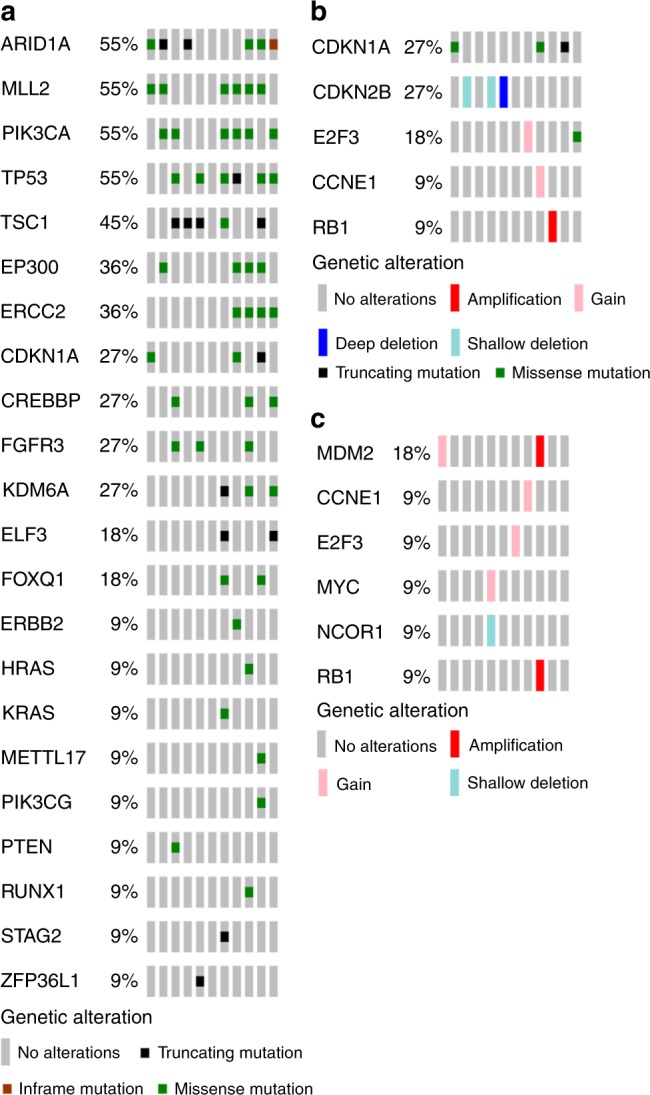
**a** Somatic mutations by patient*. **b** Copy number alterations by patient*. **c** All cell cycle pathway alterations found in study patients. *Includes all significantly mutated or altered genes identified in TCGA^4^

Sequencing data were available on 11 of the 12 enrolled patients. The most frequently observed somatic mutations were *ARID1A*, *MLL2*, *PIK3CA*, and *TP53* (55% of patients for each). Other common mutations included *TSC1*, *EP300*, and *ERCC2* (Fig. 2a). There were no patients with *CDKN2A* deletions (Fig. 2b). Alterations in the *PI3K* pathway were common with 82% of patients having a somatic mutation in either *PIK3CA* or *TSC1*. Although 82% of patients also had alterations in cell cycle genes (Fig. 2c), only 36% of patients had alterations that would predict sensitivity to palbociclib (Table 3). The two patients who responded to treatment did not harbor alterations predicted to confer sensitivity.

**Table 3:** Cell cycle pathway alterations found in study patients by IHC and next-generation sequencing

ID^a^	RB IHC	p16 IHC	Gene	Alteration	Predicted sensitivity to palbociclib	PFS4
1	3+ in 20% 2+ in 40% 1+ in 30% 0+ in 10%	3+ staining in 30%	*CDKN1A* *CDKN1A*	Asp28Gly Pro4Leu	Resistant Resistant	No
2	3+ in 10% 2+ in 20% 1+ in 20% 0+ in 50%	No staining	*CDKN2B*	Shallow deletion	Sensitive	No
3	3+ in 30% 2+ in 40% 1+ in 30%	No staining	None^a^			No
4	2+ in 30% 1+ in 40% 0+ in 30%	No staining	*CDKN2B*	Shallow deletion	Sensitive	No
5	2+ in 70% 1+ in 20% 0+ in 10%	No staining	*CDKN2B*	Deep deletion	Sensitive	No
6	2+ in 10% 1+ in 80% 0+ in 10%	No staining	None^a^			No
7	3+ in 30% 2+ in 40% 1+ in 30%	No staining	*E2F3*	Gain	Resistant	No
8	2+ in 30% 1+ in 70%	No staining	*CCNE1* *CDKN1A* *CDKN1A*	Gain Asp28Gly Pro4Leu	Resistant Resistant Resistant	Yes
9	2+ in 30% 1+ in 60% 0+ in 10%	1 + staining in 90%	*RB1*	Amplification	N/A	No
10	2+ in 50% 1+ in 30% 0+ in 20%	No staining	*CDKN1A*	FS111	Resistant	Yes
11	3+ in 20% 2+ in 20% 1+ in 10%	No staining	*E2F3*	Asp389Asn	Resistant	No
12	3+ in 10% 2+ in 30% 1+ in 30% 0+ in 30%	No staining	Unknown^a^			No

^a^Patients 3 and 6 did not have any cell cycle alterations, patient 12 did not have adequate tissue for sequencing

## Discussion

Single agent palbociclib did not demonstrate meaningful activity in molecularly selected patients with platinum-treated UC. Palbociclib has been shown to be active in some cancers with *CDKN2A* loss, although impressive single agent efficacy is limited to case reports and it is in general primarily cytostatic.^14^ Preclinical data support the potential for benefit in cancers with Rb pathway alterations,^15^ but clinical trials in liposarcoma,^16^ ovarian cancer,^17^ nonsmall cell lung cancer,^18^ and colon cancer^19^ have demonstrated stable disease in a subset of patients with few tumour responses. Our study similarly showed a minority of patients had stable disease without any tumour responses.

Despite the limited efficacy seen in our study with single agent CDK4/6 inhibition, combination therapy could be considered for further investigation. Heilmann et al. demonstrated that despite loss-of-function CDKN2A mutations in approximately 80% of sporadic pancreatic ductal adenocarcinomas, these tumours are inherently resistant to CDK4/6 inhibition, but that this resistance can be overcome with the addition of mTOR inhibition.^20^ In addition, single agent PI3K inhibitors have had only modest activity across cancers, but laboratory studies show that combined CDK4/6-PI3K inhibition can synergistically reduce cell viability.^21^ Interestingly, the patients in our study demonstrated a surprising number of alterations in the PI3K/mTOR pathway, perhaps suggesting a mechanism of resistance. It is likely, however, that tumour resistance to palbociclib occurs by multiple mechanisms, as evidenced by the breast cancer experience with the PALOMA-3 trial, which show no differential sensitivity to palbociclib based on PIK3CA mutation status.^22^ Additionally, the efficacy of CDK4/6 inhibitors in breast cancer occurred in combination with hormonal therapy, potentially highlighting the need to investigate combination therapy in UC.

Palbociclib had a tolerable safety profile in our study, with predictable and manageable AEs. Hematologic toxicity was common but only one patient required dose reduction. Neutropenia occurred frequently, but as seen in breast cancer, did not result in any episodes of febrile neutropenia. Anemia was slightly more common in our study than has been previously seen in breast cancer, which may be expected based on the patient population enrolled on this study compared to the patients typically enrolled on breast cancer clinical trials.

The lack of clinical efficacy in our trial could suggest that our inclusion criteria did not select for the patients most likely to receive benefit from palbociclib. In the PALOMA-1 trial of hormone-receptor-positive breast cancer that treated women with palbociclib plus letrozole or letrozole alone, the demonstrated improvement in PFS with palbociclib was less pronounced in the molecularly selected cohort 2 compared with the unselected cohort 1 patients, despite the presence of alterations predictive of CDK4/6 inhibitor response in cohort 2. In that trial, molecular selection was slightly different than the current study and included amplification of cyclin D1 (CCND1), loss of CDKN2A, or both. The authors postulated that relative amount of Rb protein could explain their results, and we therefore include Rb positivity by IHC as required eligibility in our trial. The results in breast cancer, however, may also simply reflect the relative importance of CDK4/6 activity in breast cancer cell proliferation independent of Rb pathway genomic alterations. UC tumours may be inherently less sensitive to the cytostatic cell cycle arrest induced by palbociclib, either due to less dependence on CDK4/6 activity or resistance mechanisms from other pathway members that are less evident in breast cancer.

The palbociclib experience in our study does highlight the difficulty in molecularly selecting patients for clinical trials via an integral biomarker at screening. Although we demonstrated the feasibility of screening patients in this setting with IHC biomarkers, IHC is a marker of protein expression and not a direct measurement of underlying molecular alterations. Molecular validation of IHC for RB in UC was performed prior to study enrollment and IHC for p16 is in widespread clinical use and correlates with CDKN2A loss reliably in other cancers.^23^ We accurately selected patients without loss of Rb (0 of 12 enrolled patients had Rb loss determined with NGS). However, there were no patients with CDKN2A loss in our study and more than the expected number of patients were eligible by our IHC screen. Prior studies in other tumours demonstrate a high sensitivity of p16 expression by IHC for underlying CKDN2A deletion, but also a substantial proportion of cases without CDKN2A deletion with p16 immunonegativity, suggesting our integral biomarkers did not select for CDKN2A deletion.^24^

Recent data confirm that CDKN2A loss is less frequent than reported in the original TCGA analysis and is now estimated at around 20–30%.^25^ Our findings suggest that the lack of p16 protein expression by IHC was not due to alterations in CDKN2A itself, but instead due to alterations in other Rb pathway members or by methylation of CDKN2A as a cause of decreased protein expression, as has been seen in other cancers.^26–29^ Alternatively, the lack of CDKN2A loss by NGS could be secondary to the noted difficulties in documenting copy number variation in single samples by NGS.^30^ This may also explain why an unexpectedly high number of patients were eligible for the study by IHC criteria. Prior studies have found that CDKN1A mutations and CDKN2A loss are mutually exclusive in bladder cancer, a finding recapitulated in our small dataset.^31^ We did not see a correlation between alterations that we predicted to confer sensitivity to palbociclib (CDKN2A inactivation, CCND1 amplification, CDKN2B inactivation) or resistance to palbociclib (Rb loss, CDKN1A inactivation, CCNE1 amplification, or E2F amplifications) and efficacy of palbociclib in our patients. Similarly, in the phase 1 trial of ribociclib (another CDK4/6 inhibitor) in solid tumours and lymphoma, CDKN2A loss by next-generation sequencing was not associated with chance of remaining on drug ≥8 weeks, although CCND1 alterations were associated with response (our study had no patients with CCND1 amplifications).^32^

One consideration is whether our trial population had more aggressive disease than a standard second-line UC population. Prior work has shown that UC tumours with CDKN2A alterations are associated with worse cancer-specific survival compared with other UCs.^33,34^ However, the median PFS in our study of 1.9 months is in keeping with other studies in this patient population and reflects the inherent aggressive nature and difficulty in treating metastatic UC after platinum-based chemotherapy. Our trial included a slightly higher percentage of upper tract UC, which could have selected for more aggressive disease, although included no patients with metastases to the liver.^35^

There are several limitations to the current study. Firstly, the integral biomarker inclusion was designed to enrich for patients more likely to respond to treatment, but does not allow for an analysis of differential response based on presence or absence of Rb pathway alterations. We are therefore unable to conclude if our inclusion criteria excluded patients that could have benefited from treatment. The only biomarker that is clinically used for CDK4/6 inhibitor treatments is hormone receptor positivity in breast cancer. Given the lack of clinical activity seen in our study, it remains unknown whether the molecular subtype of UC (i.e., basal vs. luminal) could also be a predictive biomarker in UC. An additional limitation is that sequencing was done on available archival tumour tissue, which was not confirmed to be prechemotherapy specimens and biopsy of metastatic lesions for genomic analyses was not required, which can influence detectable alterations.

In conclusion, palbociclib did not demonstrate significant activity in molecularly selected patients with platinum-refractory metastatic UC. Further development of palbociclib in UC could include combination regimens with PI3K pathway inhibitors. Additionally, if palbociclib is developed further in UC, we do not recommend IHC-based molecular selection of included patients.

## Data Availability

The clinical data generated during and/or analysed during the current study are available at https://clinicaltrials.gov/ct2/show/NCT02334527. Other datasets generated during and/or analysed during the current study are available in this article or from the corresponding author upon reasonable request.

## References

[1] Choudhury NJ (2016). Afatinib activity in platinum-refractory metastatic urothelial carcinoma in patients with ERBB alterations. J. Clin. Oncol..

[2] Petrylak DP (2017). Ramucirumab plus docetaxel versus placebo plus docetaxel in patients with locally advanced or metastatic urothelial carcinoma after platinum-based therapy (RANGE): a randomised, double-blind, phase 3 trial. Lancet.

[3] Cancer Genome Atlas Research Network. (2014). Comprehensive molecular characterization of urothelial bladder carcinoma. Nature.

[4] Robertson AG (2017). Comprehensive molecular characterization of muscle-invasive bladder cancer. Cell.

[5] Garnett MJ (2012). Systematic identification of genomic markers of drug sensitivity in cancer cells. Nature.

[6] Finn RS (2015). The cyclin-dependent kinase 4/6 inhibitor palbociclib in combination with letrozole versus letrozole alone as first-line treatment of oestrogen receptor-positive, HER2-negative, advanced breast cancer (PALOMA-1/TRIO-18): a randomised phase 2 study. Lancet Oncol..

[7] Hortobagyi GN (2016). Ribociclib as first-line therapy for HR-positive, advanced breast cancer. N. Engl. J. Med..

[8] Damrauer JS (2014). Intrinsic subtypes of high-grade bladder cancer reflect the hallmarks of breast cancer biology. Proc. Natl. Acad. Sci. USA.

[9] Choi W (2014). Identification of distinct basal and luminal subtypes of muscle-invasive bladder cancer with different sensitivities to frontline chemotherapy. Cancer Cell.

[10] Gallagher DJ, Milowsky MI, Bajorin DF (2008). Advanced bladder cancer: status of first-line chemotherapy and the search for active agents in the second-line setting. Cancer.

[11] El-Naggar AK, Westra WH (2012). p16 expression as a surrogate marker for HPV-related oropharyngeal carcinoma: a guide for interpretative relevance and consistency. Head Neck.

[12] Brat DJ (2015). Template for reporting results of biomarker testing of specimens from patients with tumours of the central nervous system. Arch. Pathol. Lab. Med..

[13] Zhao X (2015). Combined targeted DNA sequencing in non-small cell lung cancer (NSCLC) using UNCseq and NGScopy, and RNA sequencing using UNCqeR for the detection of genetic aberrations in NSCLC. PLoS ONE.

[14] Elvin JA (2017). Clinical benefit in response to palbociclib treatment in refractory uterine leiomyosarcomas with a common CDKN2A alteration. Oncologist.

[15] Tanaka T (2017). The efficacy of the cyclin-dependent kinase 4/6 inhibitor in endometrial cancer. PLoS ONE.

[16] Dickson MA (2013). Phase II trial of the CDK4 inhibitor PD0332991 in patients with advanced CDK4-amplified well-differentiated or dedifferentiated liposarcoma. J. Clin. Oncol..

[17] Konecny GE (2016). A multicenter open-label phase II study of the efficacy and safety of palbociclib a cyclin-dependent kinases 4 and 6 inhibitor in patients with recurrent ovarian cancer. J. Clin. Oncol..

[18] Gopalan PK (2014). A phase II clinical trial of the CDK 4/6 inhibitor palbociclib (PD 0332991) in previously treated, advanced non-small cell lung cancer (NSCLC) patients with inactivated CDKN2A. J. Clin. Oncol..

[19] O’Hara MH (2015). Phase II pharmacodynamic trial of palbociclib in patients with KRAS mutant colorectal cancer. J. Clin. Oncol..

[20] Heilmann AM (2014). CDK4/6 and IGF1 receptor inhibitors synergize to suppress the growth of p16INK4A-deficient pancreatic cancers. Cancer Res..

[21] Vora SR (2014). CDK 4/6 inhibitors sensitize PIK3CA mutant breast cancer to PI3K inhibitors. Cancer Cell.

[22] Cristofanilli M (2016). Fulvestrant plus palbociclib versus fulvestrant plus placebo for treatment of hormone-receptor-positive, HER2-negative metastatic breast cancer that progressed on previous endocrine therapy (PALOMA-3): final analysis of the multicentre, double-blind, phase 3 randomised controlled trial. Lancet Oncol..

[23] Mahajan A (2016). Practical issues in the application of p16 immunohistochemistry in diagnostic pathology. Hum. Pathol..

[24] Purkait S (2013). CDKN2A deletion in pediatric versus adult glioblastomas and predictive value of p16 immunohistochemistry. Neuropathology.

[25] Lerner SP (2017). Comprehensive molecular characterization and analysis of muscle-invasive urothelial carcinomas. J. Clin. Oncol..

[26] Lim AM (2014). Differential mechanisms of CDKN2A (p16) alteration in oral tongue squamous cell carcinomas and correlation with patient outcome. Int. J. Cancer.

[27] Chapman EJ, Harnden P, Chambers P, Johnston C, Knowles MA (2005). Comprehensive analysis of CDKN2A status in microdissected urothelial cell carcinoma reveals potential haploinsufficiency, a high frequency of homozygous co-deletion and associations with clinical phenotype. Clin. Cancer Res..

[28] Kurakawa E (2001). Hypermethylationofp16(INK4a) and p15(INK4b) genes in non-small cell lung cancer. Int. J. Oncol..

[29] Attri J, Srinivasan R, Majumdar S, Radotra BD, Wig J (2005). Alterations of tumour suppressor gene p16INK4a in pancreatic ductal carcinoma. Bmc Gastroenterol..

[30] Ulahannan D, Kovac MB, Mulholland PJ, Cazier JB, Tomlinson I (2013). Technical and implementation issues in using next-generation sequencing of cancers in clinical practice. Br. J. Cancer.

[31] Cazier JB (2014). Whole-genome sequencing of bladder cancers reveals somatic CDKN1A mutations and clinicopathological associations with mutation burden. Nat. Commun..

[32] Infante JR (2016). A phase I study of the cyclin-dependent kinase 4/6 inhibitor ribociclib (LEE011) in patients with advanced solid tumours and lymphomas. Clin. Cancer Res..

[33] Kim PH (2015). Genomic predictors of survival in patients with high-grade urothelial carcinoma of the bladder. Eur. Urol..

[34] Korkolopoulou P (2001). Prognostic implications of aberrations in p16/pRb pathway in urothelial bladder carcinomas: a multivariate analysis including p53 expression and proliferation markers. Eur. Urol..

[35] Akdogan B (2006). Prognostic significance of bladder tumour history and tumour location in upper tract transitional cell carcinoma. J. Urol..

